# End-of-life practices in Danish ICUs: development and validation of a questionnaire

**DOI:** 10.1186/1471-2253-12-16

**Published:** 2012-08-01

**Authors:** Hanne Irene Jensen, Jette Ammentorp, Mogens Erlandsen, Helle Ørding

**Affiliations:** 1 Department of Anaesthesiology, Vejle Hospital, Vejle, Denmark; 2 Health Services Research Unit, Lillebaelt Hospital/IRS University of Southern, Kolding, Denmark; 3 Department of Biostatistics, Aarhus University, Aarhus, Denmark

## Abstract

**Background:**

Practices for withholding or withdrawing therapy vary according to professional, cultural and religious differences. No Danish-validated questionnaire examining withholding and withdrawing practices exists, thus the aim of this study was to develop and validate a questionnaire for surveying the views of intensive care nurses, intensivists, and primary physicians regarding collaboration and other aspects of withholding and withdrawing therapy in the ICU.

**Methods:**

A questionnaire was developed on the basis of literature, focus group interviews with intensive care nurses and intensivists, and individual interviews with primary physicians. The questionnaire was validated in the following 3 phases: a qualitative test with 17 participants; a quantitative pilot test with 60 participants; and a survey with 776 participants. The validation process included tests for face and content validity (by interviewing participants in the qualitative part of the pilot study), reliability (by assessing the distribution of responses within the individual response categories), agreement (by conducting a test-retest, evaluated by paired analyses), known groups’ validity (as a surrogate test for responsiveness, by comparing two ICUs with a known difference in end-of-life practices), floor and ceiling effect, and missing data.

**Results:**

Face and content validity were assessed as good by the participants in the qualitative pilot test; all considered the questions relevant and none of the participants found areas lacking. Almost all response categories were used by the participants, thus demonstrating the questionnaires ability to distinguish between different respondents, agreement was fair (the average test-retest agreement for the Likert scale responses was 0.54 (weighted kappa; range, 0.25-0.73), and known groups’ validity was proved by finding significant differences in level of satisfaction with interdisciplinary collaboration and in experiences of withdrawal decisions being unnecessarily postponed. Floor and ceiling effect was in accordance with other questionnaires, and missing data was limited to a range of 0-7% for all questions.

**Conclusions:**

The validation showed good and fair areas of validity of the questionnaire. The questionnaire is considered a useful tool to assess the perceptions of collaboration and other aspects of withholding and withdrawing therapy practices in Danish ICUs amongst nurses, intensivists, and primary physicians.

## Background

Whether or not to withhold or withdraw therapy is a common issue in intensive care units (ICUs), as approximately 60–90% of deaths in Western ICUs occur after therapy has been withheld or withdrawn [1,2]. Withholding therapy is defined as a decision not to start or increase a life-sustaining intervention and withdrawing therapy as a decision to actively stop a life-sustaining intervention presently being given [2].

The parties involved in the decisions might not assess the situation in the same way [3-5]; among the healthcare professionals, nurses normally are the first to find therapy futile [4-6]. Practices for withholding or withdrawing therapy also vary according to cultural and religious differences [2,7-9]. In a joint European study examining end-of-life practices in ICUs [2], results from two Danish university affiliated ICUs were included [10]. Apart from this study, limited research on withholding or withdrawing therapy in Danish ICUs has been published.

A questionnaire survey was designed to determine the views of Danish intensive care nurses, intensivists, and primary physicians on withholding and withdrawal of therapy practices. As no validated Danish questionnaire exists, the aim of this study was to develop and validate a questionnaire for surveying the views of intensive care nurses, intensivists, and primary physicians regarding collaboration and other aspects of withholding and withdrawing therapy in the ICU.

## Methods

### Development of the questionnaire

#### Interviews

On the basis of the extant literature, a semi-structured interview guide was developed focusing on the following three main areas: 1) perceptions of the conditions which could/should induce considerations about withholding or withdrawing therapy in the ICU; 2) the challenges which were experienced regarding withholding or withdrawing therapy; and 3) the perception of what characterizes “good” and “poor” decision processes regarding withdrawal of therapy. In order to ensure that all issues were identified which were assessed as important by the participants, the participants were finally asked: “Are there other important areas regarding end-of-life decisions we have not talked about?”

Four focus group interviews, two with nurses and two with intensivists, were conducted (between 4 and 6 participants in each group; 21 in total). The participants were randomly selected between all staff from 2 ICUs from different hospitals in the study region (ICU A and B)**,** with staff experience taken into account, so all groups consisted of staff with short, medium, and long experiences from the ICU. The nurses were all females, whereas there were 4 female and 6 male intensivists. The intensivists included anaesthesiologists with the ICU as the main workplace and anaesthesiologists who only worked in the ICU on shifts.

From the specialities with the largest percentage of patients admitted to the ICU, primary physicians providing care for patients in the hospital prior to ICU transfer were included. For each ICU, four primary physicians were approached. The physicians were identified by the head of department, the senior secretary, or by the ICU. All primary physicians (4 females and 4 males) agreed to participate in a semi-structured, individual interview based on the same interview guide.

Both focus groups and individual interviews were audio-taped, verbally-transcribed, analysed, and the main themes were extracted [11,12]. The themes included collaborative issues, changing of withholding decisions which were already made, and unnecessary postponement of withdrawing therapy decisions. All three groups of staff agreed that the main challenge was the process of making a decision of withholding or withdrawing therapy, whereas care given to patients and relatives in whom therapy was withdrawn was found to be easy and good.

#### Questionnaire

Based on the extant literature, focus groups, and individual interviews, a questionnaire was developed in accordance with the recommendations for questionnaire design by A. Bowling [13]. In the initial process, questions were formulated, assessed, refined, or rejected in collaboration with a team of teachers in questionnaire methodology, quality managers, and intensive care nurses and physicians.

The pilot questionnaire consisted of 39 questions. Apart from questions on background characteristics, the questionnaire had 4 questions regarding reasons for withholding or withdrawing therapy, 2 questions on the process of admitting patients to the ICU, 12 questions on the decision making process regarding withholding or withdrawing therapy, 7 questions on collaboration, 3 questions on documentation, and 4 questions on care for patients and relatives in whom therapy had been withdrawn.

Although the questions were grouped by main themes, the questions were all single-item questions (each question covering a specific area with no automatic correlation expected with other question). Therefore, none of the questions permitted sum-scores [14].

The questions consisted of two types. The first type of question had responses on two different, four- or five-point Likert scales (“very satisfactory”, ”satisfactory”, ”less satisfactory”, and ”unsatisfactory”, or “always”, “often”, “sometimes”, “rarely”, and “never”, both with a “don’t know” option). The second type of question was mostly used to explain reasons for a type 1 question (“If you experience that this is happening, what are the reasons in your opinion?”) or to state recommendations, and these questions had multiple response options. For most of the questions, comments could be added.

The questionnaires were identical for the three staff groups, with the exception of a few questions about background characteristics, and a question for the primary physicians about how often they had patients they wanted to transfer to the ICU, but who were refused by the intensivists.

### Validation process

**Figure 1:**
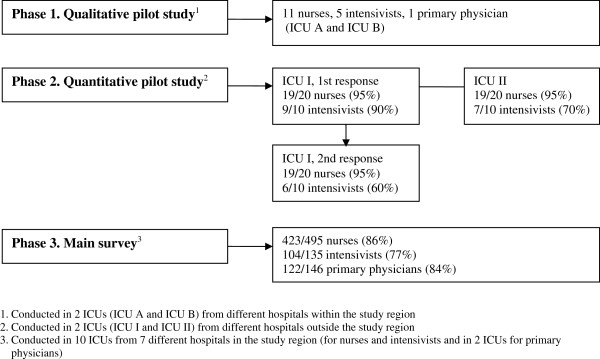
Participants and response rates in the validation study and the main survey.

The validation process consisted of the following 3 phases: a qualitative test with 17 participants from ICU A and B (from different hospitals within the study region); a quantitative pilot test with 60 participants from ICU I and II (from different hospitals outside the study region); and a survey with 776 participants from 10 ICUs (from all 7 hospitals in the study region except for primary physicians who came from only 2 of the ICUs) (Figure 1).

The aim of the validation process was to test for:

1. Face validity (does the questionnaire “look like" it is going to measure what it is supposed to measure?) and content validity (do the questions reflect areas that are essential/useful to clarify the purpose of the study?) [15]. Both face and content validity were examined by interviewing pilot study participants after they had responded to the questionnaire. The participants were asked about their general perception of the questionnaire, how they had understood the individual questions, if areas of importance in regard to the subject of the questionnaire were missing, and if any of the questions in their opinion were irrelevant.

2. Reproducibility, which includes reliability and agreement [15]. Reliability is here understood as the questionnaires ability to distinguish between different respondents. If all survey participants respond identically to the individual items, reliability will be low. Reliability is here examined by looking at the distribution of responses. Agreement is understood as consistency in a test-retest, and it is in this study examined with a weighted kappa analysis of responses provided by the same participants within a two week period.

3. Responsiveness, which means the questionnaires ability to detect improvements [15]. As no intervention was tested in the pilot study, known groups’ validity was used as a surrogate concept. Whereas ICU I had no special focus on end-of-life practices, ICU II had focused intensively on end-of-life issues and had developed and implemented guidelines for withdrawal of therapy. Known groups’ validity was tested based on the hypothesis that ICU II would be more satisfied with collaboration, less frequently experience withdrawing of therapy being unnecessarily postponed, and find care of dying patients more satisfactory compared to ICU I. We hypothesised that if the instrument’s ability to detect a difference between the two ICUs was high, the instrument would presumably also be able detect improvements within the individual ICU.

4. Floor/ceiling effect. A high floor and/or ceiling effect may reduce reliability and prevent the ability of the instrument to detect improvement because substantial samples of respondents have already reached the lowest/highest score [15].

5. Missing data. The data was analysed to assess whether or not specific questions had a high percentage of missing data or the percentage of missing data increased in the last questions, indicating that the questionnaire was too long.

### Survey

#### Qualitative pilot test

The questionnaire was first tested qualitatively for face and content validity. The test was conducted with 11 nurses and 5 intensivists from ICU A and B, and 1 primary physician affiliated with ICU A.

#### Quantitative pilot test

The questionnaire was adjusted based on results from the qualitative pilot study, then tested quantitatively in 2 ICUs from a region not part of the main study. In ICU I, 20 nurses and 10 physicians received the questionnaire twice with a 2-week interval. In ICU II another 20 nurses and 10 physicians received the questionnaire once. In both ICUs, all participants were selected by the charge nurse. The participants were asked to comment on relevance and phrasing of questions.

#### Main survey

After the pilot study, the questionnaire was used in a survey including 495 nurses and 135 intensivists from 10 ICUs and 146 primary physicians from 2 ICUs [16]. Nurses and intensivists who had been in the ICU for at least 5 months and primary physicians who had attended their patients in the ICU during the last 5 months were eligible for participation in the study. All participants received the questionnaire at their place of employment. Two reminders were sent. The study was conducted in April-June 2010 for nurses and intensivists and in June-August 2010 for primary physicians. Results from the main survey, including a copy of the questionnaire as a supplemental file, are published [16].

### Data analysis

All data were double-entered in EpiData (version 3.1), and statistical analyses were performed using STATA 10.1. A chi-square test and Mann–Whitney *U* test were used to compare background characteristics. Descriptive statistics were used to examine missing data, and the floor and ceiling effect. The weighted kappa and Wilcoxon signed-ranked test were used to test for agreement [17]. Known groups’ validity was tested by a comparison between ICU I and ICU II, using the Mann–Whitney *U* test. The level of significance was set at a p < 0.05.

Interview data and questionnaire comments were analysed using the meaning condensating method introduced by Kvale [11] and content analysis [12].

### Ethics

According to Danish law, the study did not require permission from the Regional Ethics Committee, as confirmed by the Regional Ethics Committee. Permission to obtain and store code lists of staff was granted from The Danish Data Protection Agency (j.o. 2009-41-3189). All Heads of Departments gave permission for their staff to take part in the survey. All participants were informed in writing of the purpose of the study, that participation was voluntary, and that responses were anonymous.

## Results

### Participants

As shown in Figure 1, response rates were 95% (38/40) for nurses and 80% (16/20) for intensivists in the quantitative pilot study.

Table 1 presents background information of the participants. There were no significant differences between the two ICUs.

**Table 1:** Background characteristics of participants in the quantitative pilot study

	**ICU I**^1^		**ICU II**^1^		**p**^**1**^
	**n**	**%**	**n**	**%**	
**Gender**	**28**		**26**		0.43
Male	5	(17.9)	7	(26.9)	
Female	23	(82.1)	19	(73.1)	
**Profession**	**28**		**26**		0.68
Physician	9	(32.1)	7	(26.9)	
Nurse	19	(67.9)	19	(73.1)	
**Age groups (years)**	**28**		**26**		0.43
< 30	2	(7.1)	6	(23.1)	
≥ 30 - < 40	8	(28.6)	6	(23.1)	
≥ 40 - < 50	12	(42.9)	8	(30.8)	
≥ 50	6	(21.4)	6	(23.1)	

1. ICUs from two hospitals outside the main study region.

2. Chi-square test was used for “Gender” and “Profession.”

Mann-Whitney *U* test was used for “Age groups.”

In the main survey, the overall response rate was 84% (649/776; 86% [423/495] for nurses, 77% [104/135] for intensivists, and 84% [122/146] for primary physicians). Background information of the participants in the main survey is described elsewhere [16].

### Face and content validity

All participants in the qualitative and quantitative pilot test considered the questions relevant and none of the participants found areas lacking; likewise, the participants had understood the questions as expected. Minor adjustments to phrasing and response options in multiple response questions were pointed out, and the questionnaire was changed accordingly.

### Comments

Approximately one-half of the respondents from the pilot study and the regional survey had used the opportunity to write comments, adding to more than 20,000 words. The comments elaborated the responses and thus elucidated how the questions had been understood. The comments indicated that the questions had been understood and responded to as expected. The exceptions were two questions in which a few respondents in the quantitative pilot test had misunderstood the conditions of the questions. In the main survey, the explanation was highlighted and a definition was added.

Examples of comments were:

To the question “In your experience, are there patients admitted to the ICU who in your opinion should not have been offered intensive therapy?”

"“A number of our intensivists find it hard to “say no” to the primary physicians, even though the patient does not completely fulfill the admission criteria” (nurse, responded “very often”)"

To the question: “Should the nurses be involved in the decision-making process regarding withholding or withdrawing therapy?”

"“Nurses have no background for assessing it professionally” (primary physician, responded “never”)"

To the question: “If it is your experience that decisions are unnecessarily postponed, what are the reasons in your opinion?”

"“Perhaps more uncertainty…. It is a hard and often definitive decision, and the patient’s condition and changes in this can be hard to predict” (intensivist, ticked “Fear of having to make decisions like these”)"

### Reliability

The responses showed variations. Almost all response categories were used in the quantitative pilot test and all response categories were used in the main survey, except one question in which “always” was not used, four questions in which “never” was not used, and two questions in which “unsatisfactory” and “very unsatisfactory” were not used.

### Agreement

The average test-retest agreement for the Likert scale responses was 0.54 (weighted kappa; range, 0.25-0.73). A significant difference was found for the following question: “satisfaction with collaboration,” p = 0.003 (participants were less satisfied in the re-test). Otherwise, no significant difference was found between the test and retest (average, p = 0.35; range, 0.06-1.00). A mean of 82% (range, 50%-100%) of those who had changed responses from test to retest within the Likert scale had moved only one “step” up or down the scale. Additionally, a mean of 5% (range, 0%-12%) had moved either to or from a “do not know” response. For multiple response questions, an average of 81% (range, 60-100%) of responses was identical between the test and retest.

### Known groups’ validity

Table 2 presents comparisons between responses from ICU I and ICU II on three main questions in which a difference would be expected due to ICU II having worked with guidelines for withdrawing therapy. In ICU II, the healthcare professionals were significantly more satisfied with collaboration and less often experienced decisions regarding withdrawal of life-sustaining therapy being unnecessarily postponed compared to ICU I. A non-significant difference was found in the perception of the care provided for dying patients.

**Table 2:** Comparison between ICU I and ICU II on main questions regarding withholding or withdrawing therapy practices

	**ICU I^1^**		**ICU II^1^**		**p^3^**
	**n^2^**	**%**	**n**^2^	**%**	
**Decisions regarding withdrawal of therapy are unnecessarily postponed**	**28**		**25**		< 0.001
Very often	1	(3.6)	0	(0)	
Often	10	(35.7)	2	(8.0)	
Sometimes	15	(53.6)	11	(44.0)	
Rarely	2	(7.1)	10	(40.0)	
Never	0	(0)	2	(8.0)	
**General satisfaction with collaboration**	**28**		**26**		< 0.001
Extrememly satisfactory	0	(0)	4	(15.4)	
Very satisfactory	5	(17.9)	13	(50.0)	
Satisfactory	12	(42.9)	7	(26.9)	
Less satisfactory	9	(32.1)	2	(7.7)	
Unsatisfactory	2	(7.1)	0	(0)	
**Care for dying patients**	**26**		**24**		0.07
Extrememly satisfactory	11	(42.3)	16	(66.7)	
Very satisfactory	14	(53.9)	8	(33.3)	
Satisfactory	1	(3.9)	0	(0)	

1. ICUs from two hospitals outside the main study region.

2. Different n due to “don’t know” responses and missing data.

3. Mann-Whitney *U* test.

### Floor and ceiling effect

The majority of participants in the qualitative pilot test considered the Likert scales too restricted. The floor/ceiling control showed that 50% of the questions had a ceiling effect (responses in the highest category; range, 19-94%) higher than the recommended 15% as a maximum [15,18], and 25% of the questions had > 50% of the responses in the highest category. No questions exceeded 15% in the floor effect. Therefore, the Likert scales were extended from four/five to six response options (e.g., always, very often, often, sometimes, rarely, and never). In the quantitative pilot study, the floor/ceiling effect was reduced, as follows: 55% of the questions had a ceiling effect of > 15% (range, 17-57%) with 10% of the questions having > 50% in the highest response category. No questions exceeded 15% in the floor effect.

The extended scale with the six response options was also used in the main survey, and the ceiling control showed 45% of the questions had > 15% in the highest category (range, 23-60%); 10% of the questions had > 50% in the highest category. No questions exceeded 15% in the floor effect.

### Missing data

In the pilot study, missing data for all questions ranged from 0 to 7%, and the last 5 questions had 4% missing data. In the main survey, missing data ranged from 0 to 5%. The last 5 questions had 3% missing data.

## Discussion

This study describes a validation process of a newly developed questionnaire.

The test-retest results were only fair [14,17], which may be due to a lack of stability of the instrument. However, the test-retest results may also be due to the small test-retest sample (which increases the statistical error on kappa, and reduces power on the Wilcoxon signed-rank test) and to the fact that many of the questions relate to experiences and attitudes regarding end-of-life issues which may be modified during a 2-week period of thinking about the issues. The changes from test to retest were mostly one step up or down the scale. Even with the small sample there was a significant difference between ICU I and ICU II, which indicates both that the questionnaire is able to detect changes, but also that the stability of the instrument is acceptable.

In the quantitative pilot test and main survey, the ceiling effect was reduced (range, 0-57/60%) compared to the qualitative pilot study (range, 0-94%). The range is consistent with well-known instruments [18], although it is not in agreement with the recommendations in the literature [15]. The questions with the highest ceiling effect were mainly questions regarding whether or not patients (if possible), relatives, primary physicians, and nurses should be or are involved in the decision process. The Danish law on patients’ rights makes it compulsory to discuss withholding or withdrawing therapy with a competent patient, and practice recommendations for withholding or withdrawing therapy state that decisions should be multidisciplinary [19-21]. Consequently, a high ceiling effect for these specific questions is desirable. The two other questions with the highest ceiling effect were questions regarding the quality of care for patients and relatives in whom therapy was withdrawn; areas the pre-survey interviewees had pointed out already had excellent quality. Even with the high ceiling effect, the hypothesis regarding staff from ICU II being more satisfied with withholding and withdrawing therapy practices was confirmed.

No specific questions had > 7% missing data, and the percentage of missing data did not increase towards the end, indicating that the questionnaire had an acceptable length.

There may well be other, unknown factors within the ICUs which could confound the differences identified in this study between ICU I and ICU II. Nevertheless, despite the small sample size significant differences were found in specific end-of-life areas where focus on end-of-life issues and guidelines for withdrawing life-sustaining therapy would be expected to have an impact. It is therefore likely that the guidelines and the general focus on end-of- life issues are major contributors to the differences between the ICUs. As such, we also believe that it may be plausible that the instrument will be able to detect improvements within the individual ICU (responsiveness). However, this needs to be tested in further research.

With respect to construct validity, understood as the extent to which an instrument measures the expected concept [15], none of the questions were indirect (e.g., a question regarding frame of mind is supposed to elucidate the level of depression). This increases the chance of the instrument actually measuring the construct the instrument is expected to measure. However, it also reduces the statistical possibilities to validate the instrument, as e.g. factor analysis based on multi-items (and sum scores) [22] is not applicable. The comments added by the respondents indicated that the questions had been understood and responded to as expected. Conducting surveys among healthcare professionals involve a fairly homogeneous group to whom end-of-life issues and concepts are well known; this also increases the chance of the instrument measuring the expected concepts.

Tests for internal consistency (the extent to which different items within the questionnaire are correlated) and criterion validity (the extent to which the instrument measures the expected concept) may also be conducted when validating questionnaire instruments [14,15,23,24]. However, this was not applicable for this instrument as it consists of single-item questions and because there is no gold standard to test the correlation against [15].

Another weakness of the validation study was the small sample size of the quantitative pilot study; it both increased the statistical error in the test-retest analysis and prevented extensive sub-analyses in the comparison between the two ICUs.

Knowledge about withholding and withdrawing therapy practices in the ICUs is important to improve practice, and a valid questionnaire survey will assist in detecting the issues where improvements are necessary. Results from the main survey were usable in both describing “state of the art” and identifying areas for improvement [16].

The validation process and the main survey were conducted using the Danish version of the questionnaire. Subsequently, the questionnaire was translated into English by the authors and the translation was corrected by a scientific English language company. As practices for withholding or withdrawing therapy vary according to cultural and religious differences, the questionnaire is not automatically transferable to other countries. However, the questionnaire may be either adjusted to national conditions or be useful as inspiration for development of national questionnaires.

## Conclusions

The tests showed both good and moderate areas of validity of the questionnaire. The questionnaire is considered a useful tool to assess perceptions of collaboration and other aspects of withholding and withdrawing therapy practices in Danish ICUs amongst nurses, intensivists, and primary physicians.

## Competing interests

The authors declare that they have no competing interests.

## Authors’ contributions

HIJ designed the study, collected and analysed the data, and drafted the manuscript. JA and HØ participated in designing the study and supervised data collection and analyses. ME participated in analysing the data. All authors read and approved the final manuscript.

## Pre-publication history

The pre-publication history for this paper can be accessed here:

http://www.biomedcentral.com/1471-2253/12/16/prepub
